# Factors influencing individual vaccine preferences for COVID‐19 in the Sunyani Municipality, Ghana: An observational study using discrete choice experiment analysis

**DOI:** 10.1002/hsr2.2263

**Published:** 2024-07-23

**Authors:** Samuel Fosu Gyasi, Williams Kumi, Charles Kwofie

**Affiliations:** ^1^ Department of Biological Science University of Energy and Natural Resources Sunyani Ghana; ^2^ Center for Research in Applied Biology (CeRAB) University of Energy of Energy and Natural Resources Sunyani Ghana; ^3^ Department of Mathematics and Statistics University of Energy and Natural Resources Sunyani Ghana

**Keywords:** conditional probit, COVID‐19, discrete choice experiment, efficacy, vaccine

## Abstract

**Background and Aims:**

There has been hesitancy among people with regard to accepting vaccines, especially that of coronavirus disease‐2019 (COVID‐19). This hesitancy is aggravated by the different vaccine alternatives available and what one considers before choosing a particular vaccine. The aim of this article was to investigate some driving factors that can influence an individual's COVID‐19 vaccine preference in the presence of other alternatives, using some specific vaccine characteristics.

**Methods:**

Discrete choice questionnaire was designed using the attributes and their corresponding levels to collect data on participants' preference for a COVID vaccine over a period of 12 Weeks in Sunyani, Ghana, with the help of an observational study design. A total of 150 participants receiving Covid‐19 vaccines at the University of Energy and Natural Resources Hospital were systematically selected and interviewed. Factors considered included: Efficacy of the vaccine, credibility of the manufacturing company, side effects of the vaccine, and availability of the vaccine. Data was analyzed using the conditional probit of the discrete choice experiment (DCE).

**Results:**

Results from the study using the conditional probit of the discrete choice experiment (DCE) showed efficacy, side effects, and availability as significant attributes for preference. However, there was no preference with respect to the credibility of the manufacturing company. In addition, vaccine availability was not a dis‐utility in comparison to the alternatives that are readily available. This disutility was however higher among males than females.

**Conclusion:**

From the study, most respondents preferred a COVID‐19 vaccine that is highly efficacious or a vaccine with milder side effect or a vaccine that may not necessarily be readily available. It was also observed that dis‐utility is higher among males when it comes to vaccine not being readily available than females as the odds of a female choosing a vaccine that is readily available is much higher compared to their males counterparts.

## 1 INTRODUCTION

The novel and highly pathogenic human coronavirus namely severe acute respiratory syndrome coronavirus 2 (SARS‐CoV‐2) causes the acute respiratory viral infection Coronavirus disease in 2019 (COVID‐19).^1^ Most often, this infection causes moderate to mild respiratory symptoms. Without any specific treatment, most people who get infected with the disease often recover. Persons with underlying diseases like chronic obstructive pulmonary disease (COPD) and the aged stand a higher risk of developing serious complications when infected.^1^ The underlying disease COPD together with reduction in the respiratory reserve manifestation of the diseases subsequently causes the respiratory capacity to deteriorate.^2^

The first case of COVID was reported in the province of Wuhan‐China in the year 2019, where scientists across the globe tried developing effective vaccines to reduce its impact. With the number of cases continuously increasing, there was the need to introduce an intervention which included wearing of face mask, physical distancing and frequent washing of hands with soap and alcoholic sanitizers. Unfortunately, these interventions were not enough to completely halt the rate of new infections. There was therefore the need to introduce a nonpharmaceutical intervention (NPI) in addition to the earlier mentioned controlled measures. Vaccinating the mass against Corona virus is one of the non‐pharmaceutical interventions (NPIs) that has been used in containing the COVID‐19 pandemic. Although the world had 62% fully vaccinated as of September 2022, this percentage in low‐income countries barely surpasses 20%.^3^

In Ghana, mass vaccination against Corona virus started in March 2021, with the use of AstraZeneca from Oxford UK which was mass produced by Serum Institute of India. This was made possible through a special arrangement by the Global Alliance for Vaccine Initiative (GAVI) and COVAX (In collaboration with World Health Organization [WHO] and 2 international groups which aims to send vaccines to developing countries).

The efficiency of controlling the outbreaks with NPIs during the initial stages of the pandemic when vaccines were not readily available together with the emergence of new viral variants, has been shown by a number of studies.^4^, ^5^, ^6^, ^7^ Second, during the early variants of the virus, studies focusing on vaccine effectiveness (VE) approved high protective levels in efficacy trials.^8^ Evidence to this was a study conducted in Chile on the effectiveness of the CoronaVac vaccine. During this study, a prospective follow‐up of group of vaccinated and non‐vaccinated individuals were carefully reviewed. The findings of the study revealed effectiveness rate of 65.9% against the coronavirus infection, 86.3% against associated deaths, and 90.3% against hospitalization.^9^

In Ghana, many individuals refused to be vaccinated for several reasons ranging from religious to unsubstantiated conspiracy theories put across by people on various social media platforms.^10^ In addition to these were varying side effects of the vaccines among the vaccinated population. These included headache, severe cold, dizziness, and redness of the site of injection, and many others.^10^ Some individuals also refused to get vaccinated with the prejudice that subsequent doses after the first shot (especially with AstraZeneca vaccine) may not be available when their second dose is due. One other critical piece of information that was missing in the policies of the manufacturing companies of the COVID‐19 vaccine was perceived to be the side effect of the vaccines. These subsequently affected the propensity to mitigate the side effects associated with the vaccines, and people automatically refused to have the vaccine administer.^10^

Besides the side effects of the vaccine, one paramount indicator that people will definitely pay attention to is how credible the manufacturing company is perceived to be in terms of drug production and its efficacy rate. Again, one crucial need of the COVID‐19 vaccine that the world is seeking is its efficacy and reliability. In this increasing pandemic, the world is in need of a reliable and efficient version of the vaccine against individuals suffering the pandemic.^11^ With COVID‐19 still being in the shadows and people refusing to take the vaccine for reasons known to them, there is a need to study what will drive a person to choose one COVID vaccine among several competing vaccines produced by the various companies. This has necessitated this study. It will be very appropriate to know what influences the choice of a COVID‐19 vaccine over another; this will also be very informative in the decision‐making of manufacturing companies.

Factors that affect one's perception and opinions about a product can be the product‐related characteristics (In our case COVID vaccine‐related characteristics), social characteristics, religious, and other characteristics (see e.g.,^12^, ^13^, ^14^, ^15^). As such several factors can influence people's preference for vaccine uptake. Aside vaccine‐specific characteristics, COVID‐19 vaccine uptake is influenced by several other factors ranging from demographic to psychological factors. Social factors such as sociodemographic characteristics, perceived risk and benefits, trust in vaccine producers, waiting time and convenience, religious and cultural beliefs, awareness and education campaigns, social influences, and peer pressure can also significantly influence the vaccine uptake when carefully addressed through tailored public health strategies. Again, another intriguing factor that can influence vaccine preference is the media coverage, news reports, social media, and public discussions about different vaccines can shape perceptions and influence preferences of people on the vaccine. Furthermore, endorsement of the vaccine by public figures, celebrities, and influential community leaders can sway individual choices and influence preference.

It is therefore evident that outside the scope of the vaccine characteristics such as efficacy of the vaccine, side effect of vaccine, credibility of the company manufacturing the vaccine, and availability of vaccine, several other factors that is not directly related to the product can influence one's preference for the product. Some studies have looked at how other characteristics that are not directly product specific characteristic such as social media, public endorsements and religious beliefs, and others can affect one's preferences (see references^13^, ^14^, ^15^). Hence our main goal and aim was to focus on only vaccine‐related characteristics that influence one's preference. These were carefully chosen in order not to over increase the choice sets as increasing the number of attributes increases the choice sets (number of questionnaires) which may lead to respondents' fatigue.

From statistical point of view, the discrete choice experiment (DCE) which is used in studying preferences or choices has barely been used in the medical field. In this study, we designed a discrete choice experiment for studying preferences between alternative available vaccines. These were adopted to answer the following research questions:

- What are the key factors that influence individuals' decision‐making process when choosing between alternative COVID‐19 vaccines?
- What role do the demographic factor such as gender play in individuals' vaccine preference and decision‐making?
- To what extent do individuals consider vaccine availability, (accessibility), in their decision‐making process?
- How does the credibility of a vaccine produce company impact on individuals' preferences for different COVID‐19 vaccines?
- How do individuals balance the benefits of individual protection against COVID‐19 with the potential side effects, when choosing between vaccines?

For ease of estimation and reduced choice sets (discrete choice questions), we assume that each attribute has two levels. Below is a brief note on the attributes considered in this study.

### 1.1 Availability of the vaccine

The “availability of the vaccine” here refers to the ease of getting the vaccine when needed. As trivial as this attribute is, it is a key factor that can influence a person to choose a particular vaccine. This is in particular important due to the fact that, for the COVID‐19 vaccine, a second dose and in some cases booster doses are required for complete protection against the disease. According to Forni et al.,^16^ one technical challenge in the midst of the pandemic is connected with the availability of these vaccines, especially in developing countries like Ghana. In our part of the world, where the vaccine is not available in large quantities as expected, people will surely consider the type of vaccine that is readily available should they agree to get vaccinated. This will increase their quest to get all the doses of the vaccine on time when it becomes necessary. Vaccine hesitancy is the delay or refusal in accepting a vaccination despite its availability during a vaccination program.^17^

### 1.2 Credibility of the manufacturing company

Due to the counterfeiting of vaccines on a global scale, one trivial factor that possibly influence people's preference is the credibility and reputation of the producers of that vaccine.^18^ According to Youssef et al.,^19^ one factor that influences the willingness of an individual to voluntarily take the COVID‐19 vaccine is the reliability of the manufacturing company. The acceptance rate of the COVID‐19 vaccine in relation to the manufacturing company increased among health care workers, who are at higher risk of the pandemic.

### 1.3 Efficacy of the vaccine

One crucial factor that influences people's preference for a vaccine is its efficacy. A controlled clinical trial is used to measure the efficacy of a vaccine by comparing the number of individuals involved in the study who subsequently developed the “outcome of interest” (usually a disease) after receiving a vaccine, with the number of individuals who received a placebo after being exposed to the same outcome. Due to the deadly nature of the COVID‐19, every rationale person who agrees to be vaccinated will consider how efficacious the vaccine is in offering the needed protection once exposed. They are more likely to opt for the most efficacious vaccine to enhance their chance of survival among several competing options in the market. According to Solis Arce,^20^ vaccine efficacy and safety, as reported by healthcare workers, could be one of the paramount reasons for hesitancy in the low middle income countries, hence the choice of this attribute.

### 1.4 Side effect of the vaccine

One other key issue that almost every potential vaccine recipient will consider in choosing a particular vaccine in the presence of other brands of the same vaccine type will be the side effects. One major concern about vaccine hesitancy, especially in developing countries like Ghana, is the side effects of the vaccine.^20^ After vaccination, most people interact among themselves and share their post vaccination experiences. The presence of adverse side effects following vaccination is enough to influence one's decision to opt for a particular vaccine and whether or not an individual accepts a vaccine.

## 2 MATERIALS AND METHODS

The method of data collection, sampling technique, sample size calculation and the theoretical framework for analysing the discrete choice questionnaires are presented in this section.

### 2.1 Data collection

Discrete choice questionnaire was designed using the attributes and their corresponding levels. These questions were used to collect data on participants' preference for a COVID vaccine in the presence of an alternative over a period of 12 weeks at Sunyani Bono Region within the middle belt of Ghana, West Africa. Systematic random sampling was used to select individuals within the hospital. The inclusion criterion is that an individual must be qualified to receive the COVID vaccine in Ghana by the Ministry of Health standards.

### 2.2 Sampling technique

The respondents (sample) for this study were selected using systematic sampling technique. This technique ensured that, in selecting individuals during the data collection, each person who visited the hospital had equal chance of being selected.

### 2.3 Sample size determination

The DCE was conducted among the indigenes in Sunyani, Bono region‐Ghana. We used stated preference surveys where data is collected experimentally rather than through observation (see Street et al.,^21^). We used hypothetical choice sets to collect the data similar to Yan et al.^22^ The lowest number of respondents for the study according to Hensher et al.^23^ is given by $N=\frac{n}{r}$. In this study, we used stated preference surveys by developing a set of hypothetical questions (for details see Hensher et al.^23^). Using the sample size calculation formula adopted by Hensher et al.,^23^ the minimum number of sample is defined as:

(1) $$n\geq \frac{q}{p\alpha^{2}}{\left[\Phi^{-1}\left(1-\frac{\alpha }{2}\right)\right]}^{2},$$

where p is the percentage of individuals having some characteristic of desirable interest and q=1−p. $\Phi^{-1}\left(1-\frac{\alpha }{2}\right)$ is the inverse of the standard normal (*z*) cumulative distribution function, and α is the error margin term. Using the rule of thumb described in Kwofie et al.,^24^ we obtain a sample size of n≥1536 keeping in mind the fact that there are only two choices A and B. Hence, we obtain the minimum number of respondents to be $N=\frac{1536}{28}=55.$ With this minimum number (55 for each option) in mind, we selected 150 respondents of which 141 were deem fit for further analysis. It is worthwhile to note that for two alternatives,^23^ assert that at least 50 decision‐makers for each alternative” is sufficient. This means that, 100 individuals are enough to conduct the study of which this we far exceeded.

### 2.4 Model specification and theoretical framework of the choice experiment

McFadden's random utility theory laid the foundation for preference modeling (see McFadden^25^). Preference modeling using discrete choice experiment relies on the idea of random utility model. In a random utility model, which forms the framework of DCE, we have that an individual's utility of any choice can be expressed mathematically as:

(2) $$U_{\mathrm{st}}=V_{\mathrm{st}}+\varepsilon_{\mathrm{st}}.$$

$U_{\mathrm{st}}$ is the inherent utility attached to alternative choice s by the t−th individual. Also, $U_{\mathrm{st}}$ is a deterministic utility while $\varepsilon_{\mathrm{st}}$ is a random error term. $V_{\mathrm{st}}$ is a deterministic variable of the alternative choice which incorporates the attributes of the alternative itself and the features of the individual. According to McFadden,^25^ some degree of randomness feeds into the choices of an individual. According to McFadden, this randomness is a utility and can be written mathematically as:

(3) $$U_{\mathrm{st}}=\beta +\sum_{a=1}^{A}\delta_{a}V_{\mathrm{sat}}+\sum_{m=1}^{M}\alpha_{m}Y_{\mathrm{mt}}+\sum_{a=1}^{A}\sum_{m=1}^{M}\beta_{\mathrm{am}}X_{\mathrm{sat}}Y_{\mathrm{mt}}+\mu_{\mathrm{st}}$$

where choice of vaccine s = [Vaccine A, Vaccine B] and *t* = 1*… M* denotes respondents, *Y* is a vector of *N* individual features and *V* is a vector of *A* attribute levels. The variable $\mu_{\mathrm{st}}\;$is a random occurrence not observed effects on individual preference. The parameter $\delta_{a}$ is the utility associated with vaccine with attribute *a* and $\beta_{\mathrm{am}}$ is the parameter that shows how this utility differs among different individuals. In this instance, it is assumed that the person chooses the vaccine that provides greater utility. For respondent *t*, the utility gain for choosing Vaccine A over B is given by;

(4) $$U_{\mathrm{Bt}}-U_{\mathrm{At}}=\sum_{a=1}^{A}\delta_{a}\left(V_{\mathrm{Bat}}-V_{\mathrm{Aat}}\right)+\sum_{a=1}^{A}\sum_{m=1}^{N}\beta_{\mathrm{am}}\left(V_{\mathrm{Bat}}-V_{\mathrm{Aat}}\right)Y_{\mathrm{mt}}+(\mu_{\mathrm{bt}}-\mu_{\mathrm{at}})$$

In theory, the random occurrence factor $\mu_{\mathrm{st}}$ contain three additive terms: individual exact component $V_{t}$, COVID vaccine choice component $e_{s}$ and true iid random variable. The individual chooses COVID vaccine A if;

(5) $$U_{\mathrm{Bt}}-U_{\mathrm{At}}>0.$$

This occurs by taking probability of the Equation (4). If the difference in utility is assumed to follow a standard normal distribution, then this probability can be described using the conditional probit model. Hence using the approach proposed by Kwofie et al.,^24^ we analyse the discrete choice responses using the conditional probit model. All statistical significance of variables in the conditional probit model are determined at a significance level of 5%.

### 2.5 Attributes and their corresponding levels

In this study, we considered Four (4) attributes of the COVID‐19 vaccine and their corresponding levels known from literature. The attributes were efficacy of the vaccine, credibility of the manufacturing company, availability of the vaccine and side effect of the vaccine. Table 1 below presents the attributes and their corresponding levels.

**Table 1:** Attributes and their corresponding levels.

Attributes	Levels
Efficacy	High, satisfactory
Credibility	High, satisfactory
Side effect	Mild, severe
Availability	Readily available, not readily available

### 2.6 Constructing the choice set experiment

The orthogonal arrays generated from the attributes and their corresponding levels were done Using SPSS. These arrays are used in obtaining the choice sets. The orthogonal design generated eight profiles and this was used to develop the choice sets. The results of the eight profiles are displayed in Table 2 below.

**Table 2:** Orthogonal arrays generated from SPSS.

Choice set	Efficacy	Credibility	Side effects	Availability
1	High	Satisfactory	Mild	Readily available
2	Satisfactory	High	Mild	Readily available
3	High	High	Mild	Not readily available
4	Satisfactory	Satisfactory	Severe	Not readily available
5	Satisfactory	Satisfactory	Mild	Not readily available
6	High	High	Severe	Not readily available
7	High	Satisfactory	Severe	Readily available
8	Satisfactory	High	Severe	Readily available

Table 3 below shows one of the choice sets (questions) presented to the respondents. It is such that, given the levels of the various attributes presented, individuals are to make a choice between vaccine A or B by ticking their preferred hypothetical vaccine.

**Table 3:** Choice set presented to respondents to make a choice.

Attributes	Vaccine A	Vaccine B
Efficacy	High	Satisfactory
Credibility	Satisfactory	High
Side Effect	Mild	Severe
Availability	Not readily available	Readily available
Which one of these COVID vaccine would you choose?	Vaccine A □	Vaccine B □

## 3 RESULTS

We now present results from the conditional probit model of the DCE. This model helps identify how each level of an attribute affects individual's preference among alternative COVID‐19 vaccines. One hundred and forty‐one (141) responses were used for the analysis. Out of this number, 47.3% were males while 52.7% were females. The Discrete choice questionnaire has 28 choice sets and as such the number of entries becomes 28*2*141 = 7896 entries. First we present the full conditional probit model result followed by the gender restricted conditional probit result.

### 3.1 Conditional probit model

We used the conditional probit model to estimate how each level of the attributes influences individuals' preference when choosing between two COVID‐19 vaccines A and B. These are hypothetical questions we ask so that given the various levels of the attributes an individual can make a choice (see e.g., Table 3). In this regard, respondents at every point were presented with two choices with differing attributes levels. Table 4 below shows the parameter estimate of the conditional probit model for the responses. As a standard way of analysis, we code the attributes as factors in *R*‐software and as a result, *R* automatically chooses one level of each attribute as the reference point for comparison. For the attributes efficacy and credibility with levels satisfactory and high, high was chosen as the reference point for both variables; for the attribute side effect, having levels mild and severe, severe was chosen as the reference point. In a similar fashion, for the attribute availability, having the levels, readily available and not readily available, *R* chose not readily available as the reference point for comparison.

**Table 4:** Parameter estimates of conditional probit model for Covid vaccine.

Attributes	Coefficient	Exp. coefficient	Standard error	*z*‐Values	*p*‐Value
Efficacy: Satisfactory	−0.3215	0.7251	0.1319	−2.437	0.015
Credibility: Satisfactory	−0.1829	0.8328	0.1348	−1.357	0.17
Side effect: Mild	0.8583	2.3591	0.1366	6.282	<0.001
Availability: Readily available	−0.2285.	0.5909	0.1370	−3.842	<0.001
Number of observations	7896				
AIC	589.7				
Likelihood ratio (*χ* ^2^)	355				
*p*‐Value	0.0000				

The probit model in Table 4 revealed that apart from Credibility of the manufacturing company, all the other variables were significant predictors of preference at a significance level of 5%. Efficacy of the vaccine being satisfactory is a dis‐utility, meaning respondents will prefer a COVID 19 vaccine that is highly efficient to satisfactorily efficient vaccines. The odds of an individual choosing COVID‐19 vaccine that is satisfactorily efficacious over highly efficacious vaccine decreases by 27.49%. This reduction is not so huge implying that there is also the likelihood that some respondents may not be too concern about high efficacy and they may be okay with efficacy that is satisfactory. There is a 100% increase in the odds of respondents choosing vaccines with mild side effect in comparison to vaccines with severe side effect when presented with both options. Surprisingly, availability of the vaccine being not readily available is not a dis utility. Respondents do not really care about COVID 19 vaccines that are readily available in comparison to those that are not readily available. The odds of an individual choosing a vaccine that is readily available in comparison to those that are not readily available decreases by 40.91%. This percentage implies that, some individuals are not really concerned about readily availability of a vaccine. However, this percentage is not close to even 50%, implying that there are still some individuals who are concerned with availability of the vaccine. A plausible reason for which individuals may not be so concerned about readily availability may be due to individual's lackadaisical attitude towards vaccination or for other reasons.

### 3.2 Restricted conditional probit model for gender

Here we now analyse the responses of males and females separately to see how gender affects vaccination preference. Tables 5 and 6 below illustrate the restricted conditional probit for females and males respectively.

**Table 5:** Restricted conditional probit model for females.

Attributes	Coefficient	Exp. coef	Standard error	*z*‐Value	*p*‐Value
Efficacy: High	1.3357	3.8026	0.7314	1.826	0.07
Credibility: high	0.5676	1.7641	0.8265	0.687	0.49
Side effect: mild	1.8519	6.3722	0.8936	2.072	0.04
Availability: readily available	−1.4307	0.2391	0.7144	−2.003	0.04
Number of observations	3976				
AIC	4251.9				
*p*‐Value	0.0000				

**Table 6:** Restricted conditional probit model for males.

Attributes	Coefficient	Exp. coef.	Standard error	*z*‐Value	*p*‐Value
Efficacy: high	−17.95	1.609e‐08	1.300	−13.81	<0.001
Credibility: high	−35.61	3.416e‐16	1.322	−26.93	<0.001
Side effect: mild	−17.67	2.123e‐08	1.300	−13.59	<0.001
Availability: readily available	−37.35	5.996e‐17	1.322	−28.25	<0.001
Number of observations	3920				
AIC	3271.8				
Likelihood ratio (*χ* ^2^)	514.461				
*p*‐Value	0.0000				

The conditional probit model in Table 5 revealed that only availability of vaccine and side effect were significant attributes for preference among females whiles all the attributes were significant among males. There is a 100% increase in the odds of females choosing vaccines with mild side effect in comparison to vaccines with severe side effect when presented with both options whiles that of males is the complete opposite. It appears the odd of a vaccine having mild side effect does not increase the chances of males choosing that vaccine. This indicates that they may be indifferent when it comes to side effect provided the vaccine has been approved scientifically. Availability of the vaccine being not readily available is not a dis utility among both males and females just like the full model. Respondents do not really care about COVID 19 vaccines that are readily available in comparison to those that are not readily available. The odds of an individual choosing a vaccine that is readily available in comparison to those that are not readily available decreases by 76.1% among females while that of males is 99.99%. This simply means that females care more about availability than males since the odds is higher for males.

## 4 DISCUSSION

As much as respondents are contemplating the preference of the COVID‐19 vaccine, manufacturing companies and scientists are also agitating about the choice of the vaccine that will receive global acceptance to minimize vaccine hesitancy. One major challenge of the COVID 19 vaccination program is vaccine hesitancy (see, references^26^, ^27^). Several factors can influence people's preference for vaccine uptake. These factors range from demographic to psychological factors. Social factors such as sociodemographic characteristics, perceived risk and benefits, trust in vaccine producers, waiting time and convenience, religious and cultural beliefs, awareness and education campaigns, social influences and peer pressure can also significantly influence the vaccine uptake. Again, media coverage, news reports, social media, and public discussions about different vaccines can shape perceptions and influence preferences of people on the vaccine. Furthermore, endorsement of the vaccine by public figures, celebrities, and influential community leaders can sway individual choices and influence preference.

However, for ease in the choice set questionnaire, four main attributes (Efficacy, credibility, side effect, and availability) were modeled to investigate how each influences the choice of COVID‐19 vaccines among individuals. To ascertain how these attributes influence individuals' preferences for the COVID‐19 vaccine in the Sunyani municipality of Ghana, the conditional probit model of the discrete choice experiment was used. From our model, the best scenario individuals may prefer in choosing one vaccine over the other is when the vaccine is highly efficacious, has mild side effect and may not necessarily be available for use. From the study, efficacy of the vaccine was a significant factor influencing one's choice for the vaccine, thus, the vaccine being satisfactorily efficacious is a dis‐utility, meaning that respondents will rather choose a vaccine that has high efficacy.

According to Davis et al.,^28^ the efficacy of COVID‐19 vaccine has a significant bearing on whether an individual will be interested in taking the vaccine or not. This could also be best explained by the fact that in the era of the global pandemic when lives of people were at risk and precious lives were been taken every second, every rational person wants to be vaccinated to increase his/her chance for survival. The study showed that individuals preferred vaccines with high efficacy over satisfactorily efficacious COVID 19 vaccines. This is consistent with the work of Davis et al.^28^ It is therefore not surprising that the COVID 19 vaccine has to go through series of clinical trials by the manufacturing companies and scientist to increase its efficacy rate and subsequently receiving global acceptance and patronage.

Side effect of the vaccine was a significant factor that influenced the choice for a vaccine. Our results showed that individuals preferred vaccines with mild side effect over those with severe side effect. However, the odds of choosing a vaccine with mild side effect was a little below 50% indicating that there is the possibility of some individuals choosing vaccine with severe side effect provided it has other desirable qualities. This confirms a survey conducted by Motta^29^ in the United states on vaccination intention. It was concluded from the work that respondents will prefer vaccine with less than 1% risk level of minor side effect. It is therefore not surprising that respondents of this study opine that vaccine with milder side effect is preferable.^29^ also confirms the effect of this attribute.

Lastly, availability of the vaccine was a significant factor that influenced individuals' choice for a vaccine. Surprisingly, individuals were not enthused about the availability of vaccine. This is inconsistent with the work of Dong et al.,^30^ who investigated preference for vaccine in China. They found out that majority of the people (80%) who participated in the survey will prefer to take the vaccination when it is readily available. A plausible reason for the difference in result could be due to the geographical location of respondents or due to the economic prowess of China, making availability a non‐existent issue in a country like Ghana. This could also be that in a developing country like Ghana where vaccines were not coming frequently as expected, respondents will definitely make non‐availability a nonexistent issue due to their own lack of desire to vaccinate. However, Cioffi et al.,^31^ suggest that there is the need to put in place special behavioral interventions to enhance the accessibility of the COVID‐19 vaccine.

## 5 CONCLUSION

This study investigated respondents' preference for the choice of COVID‐19 vaccine in University hospital‐Sunyani municipality‐Ghana, using a discrete choice experiment. Although several factors such as endorsement by public figures, demographic factors, media sensitization etc. can influence vaccine uptake, we investigated 4 attributes and their corresponding levels, we measure how each attribute affected an individual's COVID‐19 vaccine preference. The four attributes and their corresponding levels were efficacy (high/satisfactory), credibility (high/satisfactory), side effect (mild/severe), and availability (readily available/not readily available). The conditional probit model of the DCE was used. The only insignificant attribute was the credibility of the manufacturing company, with the remaining attributes being significant. From the study, most respondents preferred a COVID‐19 vaccine that is highly efficacious or a vaccine with milder side effect or a vaccine that may not necessarily be readily available. It was also observed that disutility is higher among males when it comes to vaccines not being readily available than females as the odds of a female choosing a vaccine that is readily available is much higher compared to their males counterparts. A combination of these reasons for their preference ensures that they choose what maximize their utility. It is therefore recommended that other DCE models like the nested logit and even the multinomial conditional probit models are used in situations where one is presented with more than two alternatives. Some more attributes such as demographic factors, media publicity, public endorsement etc. and levels with more than two categories could also be considered although this may lead to increased choice sets which may lead to fatigue among respondents. As further future work, we recommend that studies that combines both vaccine‐related characteristics and other non‐vaccine related characteristics such as social media and others are investigated concurrently to investigate vaccine uptake and hesitancy using discrete choice experiment.

## AUTHOR CONTRIBUTIONS

**Samuel Fosu Gyasi**: Writing—review & editing; writing—original draft; supervision; resources; investigation; conceptualization. **Williams Kumi**: Data curation; formal analysis; conceptualization; investigation; writing—original draft. **Charles Kwofie**: Methodology; formal analysis; conceptualization.

## CONFLICT OF INTEREST STATEMENT

The authors declare no conflict of interest.

## ETHICS STATEMENT

Ethical clearance with reference number CHRE/AP/186/022 was obtained from the committee for human research and ethics, University of Energy and Natural Resources.

## TRANSPARENCY STATEMENT

The lead author Charles Kwofie affirms that this manuscript is an honest, accurate, and transparent account of the study being reported; that no important aspects of the study have been omitted; and that any discrepancies from the study as planned (and, if relevant, registered) have been explained.

## Data Availability

The data that support the findings of this study are available from the corresponding author upon reasonable request. Data is available upon request from the corresponding author.

## References

[1] Fekete M , Horvath A , Santa B . Covid‐19 vaccination coverage in patients with chronic obstructive pulmonary disease–a cross‐sectional study in Hungary. Vaccine. 2023;41(1):193‐200. 36424256 10.1016/j.vaccine.2022.11.020 PMC9671791

[2] Varga JT . Smoking and pulmonary complications: respiratory prehabilitation. J Thorac Dis. 2019;11(suppl 5):S639‐S644. 31080640 10.21037/jtd.2018.12.11 PMC6503270

[3] Batista C , Hotez P , Amor YB . The silent and dangerous inequity around access to covid‐19 testing: a call to action. eClinicalMedicine. 2022;43:101230. 34927038 10.1016/j.eclinm.2021.101230 PMC8668028

[4] Bo Y , Guo C , Lin C . Ef‐ fectiveness of non‐pharmaceutical interventions on covid‐19 transmission in 190 countries from 23 January to 13 April 2020. Int J Infect Dis. 2021;102:247‐253. 33129965 10.1016/j.ijid.2020.10.066 PMC7598763

[5] Ge Y , Zhang WB , Liu H . Impacts of worldwide individual non‐pharmaceutical interventions on covid‐19 transmission across waves and space. Int J Appl Earth Obs Geoinform. 2022;106:102649. 10.1016/j.jag.2021.102649 PMC8666325 35110979

[6] Iezadi S , Gholipour K , Azami‐Aghdash S . Effectiveness of non‐pharmaceutical public health interventions against covid‐19: a systematic review and meta‐analysis. PLoS One. 2021;16(11):e0260371. 34813628 10.1371/journal.pone.0260371 PMC8610259

[7] Stokes J , Turner AJ , Anselmi L , Morciano M , Hone T . The relative effects of non‐pharmaceutical interventions on wave one covid‐19 mor‐ tality: natural experiment in 130 countries. BMC Public Health. 2022;22(1):1113. 35659646 10.1186/s12889-022-13546-6 PMC9165709

[8] Chuenkitmongkol S , Solante R , Burhan E . Expert review on global real‐world vaccine effectiveness against sars‐cov‐2. Expert Rev Vaccines. 2022;21(9):1255‐1268. 35748494 10.1080/14760584.2022.2092472

[9] Paternina‐Caicedo A , Jit M , Alvis‐Guzmán N . Effectiveness of coronavac and bnt162b2 covid‐19 mass vaccination in Colombia: a population‐based cohort study. Lancet Reg Health‐Am. 2022;12:100296. 35791428 10.1016/j.lana.2022.100296 PMC9246705

[10] Akther T , Nur T . A model of factors influencing covid‐19 vaccine accep‐ tance: a synthesis of the theory of reasoned action, conspiracy theory belief, awareness, perceived usefulness, and perceived ease of use. PLoS One. 2022;17(1):e0261869. 35020764 10.1371/journal.pone.0261869 PMC8754289

[11] Kaur RJ , Dutta S , Bhardwaj P . Adverse events reported from covid‐19 vaccine trials: a systematic review. Indian J Clin Biochem. 2021;36(4):427‐439. 33814753 10.1007/s12291-021-00968-z PMC7997788

[12] Wiese M , Akareem HS . Determining perceptions, attitudes and behaviour towards social network site advertising in a three‐country context. J Market Manag. 2020;36(5‐6):420‐455.

[13] Ahmad Rizal AR , Nordin SM , Ahmad WFW , Ahmad Khiri MJ , Hussin SH . How does social media influence people to get vaccinated? Rhe elaboration likelihood model of a person's attitude and intention to get COVID‐19 vaccines. Int J Environ Res Public Health. 2022;19(4):2378. 35206563 10.3390/ijerph19042378 PMC8872449

[14] Chadwick A , Kaiser J , Vaccari C . Online social endorsement and Covid‐19 vaccine hesitancy in the United Kingdom. Social Media+ Soc. 2021;7(2):205630512110088.

[15] Alam F , Tao M , Rastogi R , Mendiratta A , Attri R . Do social media influencers influence the vaccination drive? An application of source credibility theory and uses and gratification theory. Technol Forecase Soc. 2024;198:122973.

[16] Forni G , Mantovani A , R. COVID‐ Commission of Accademia Nazionale dei Lincei . Covid‐19 vaccines: where we stand and challenges ahead. Cell Death Differ. 2021;28(2):626‐639. 33479399 10.1038/s41418-020-00720-9 PMC7818063

[17] El‐Elimat T , AbuAlSamen MM , Almomani BA , Al‐Sawalha NA and Alali FQ . Acceptance and attitudes toward covid‐19 vaccines: a cross‐sectional study from Jordan. PLoS One. 2021;16(4):e0250555. 33891660 10.1371/journal.pone.0250555 PMC8064595

[18] Jarrett S , Wilmansyah T , Bramanti Y . The role of manufacturers in the implementation of global traceability standards in the supply chain to combat vaccine counterfeiting and enhance safety monitoring. Vaccine. 2020;38(52):8318‐8325. 33199075 10.1016/j.vaccine.2020.11.011 PMC7727743

[19] Youssef D , Abou‐Abbas L , Berry A , Youssef J and Hassan H . De‐ terminants of acceptance of coronavirus disease‐2019 (covid‐19) vaccine among Lebanese health care workers using health belief model. PLoS One. 2022;17(2):e0264128. 35192664 10.1371/journal.pone.0264128 PMC8863223

[20] Solís Arce JS , Warren SS , Meriggi NF . Covid‐19 vaccine acceptance and hesitancy in low‐and middle‐income coun‐ tries. Nature Med. 2021;27(8):1385‐1394. 34272499 10.1038/s41591-021-01454-y PMC8363502

[21] Street DJ , Burgess L and Louviere JJ . Quick and easy choice sets: constructing optimal and nearly optimal stated choice experiments. Int J Res Market. 2005;22(4):459‐470.

[22] Yan X , Levine J , Zhao X . Integrating ridesourcing services with public transit: an evaluation of traveler responses combining revealed and stated preference data. Transp Res Part C: Emerg Technol. 2019;105:683‐696.

[23] Hensher DA , Rose JM , Greene WH . Applied Choice Analysis: A Primer. Cambridge University Press, 2005.

[24] Kwofie C , Yormekpe DD , Mensah SO , Botchway P . Choosing between alternative motor insurance policies: a discrete choice experiment. In J Math Math Sci. 2018;38:4587987.

[25] McFadden D . Frontiers in econometrics, chapter conditional logit analysis of qualita‐ tive choice behavior. Academic Press; 1974.

[26] Freeman D , Loe BS , Chadwick A . Covid‐19 vaccine hesitancy in the UK: the Oxford coronavirus explanations, attitudes, and narratives survey (oceans) ii. Psychol Med. 2022;52(14):3127‐3141. 33305716 10.1017/S0033291720005188 PMC7804077

[27] Wu J , Li Q , Silver Tarimo C . Covid‐19 vaccine hesitancy among Chinese population: a large‐scale national study. Front Immunol. 2021;12:4833. 10.3389/fimmu.2021.781161 PMC8666422 34912346

[28] Davis CJ , Golding M and McKay R . Efficacy information influences intention to take covid‐19 vaccine. Br J Health Psychol. 2022;27(2):300‐319. 34250684 10.1111/bjhp.12546 PMC8420419

[29] Motta M . Can a covid‐19 vaccine live up to Americans' expectations? A conjoint analysis of how vaccine characteristics influence vaccination intentions. Soc Sci Med. 2021;272:113642. 33414031 10.1016/j.socscimed.2020.113642 PMC7832269

[30] Dong D , Xu RH , Wong EL . Public preference for covid‐19 vaccines in China: a discrete choice experiment. Health Expect. 2020;23(6):1543‐1578. 33022806 10.1111/hex.13140 PMC7752198

[31] Cioffi CC , Kosty D , Nachbar S , Capron CG , Mauri‐ cio AM , Tavalire HF . Covid‐19 vaccine deliberation among people who inject drugs. Drug Alcohol Depend Rep. 2022;3:100046. 35345466 10.1016/j.dadr.2022.100046 PMC8942572

